# The Challenges of a Children’s Hospital during the COVID-19 Pandemic: The Pediatric Surgeon’s Point of View

**DOI:** 10.3390/pediatric12030025

**Published:** 2020-11-12

**Authors:** Gloria Pelizzo, Sara Costanzo, Luciano Maestri, Giorgio Giuseppe Orlando Selvaggio, Andrea Pansini, Gian Vincenzo Zuccotti, Elena Zoia, Giuseppe De Filippis, Alessandro Visconti, Valeria Calcaterra

**Affiliations:** 1 Department of Pediatric Surgery, “V. Buzzi” Children’s Hospital, University of Milan, 20157 Milan, Italy; sara.costanzo@asst-fbf-sacco.it (S.C.); luciano.maestri@asst-fbf-sacco.it (L.M.); giorgio.selvaggio@asst-fbf-sacco.it (G.G.O.S.); andrea.pansini@asst-fbf-sacco.it (A.P.); 2 Department of Biomedical and Clinical Sciences "L. Sacco", University of Milan, 20157 Milano, Italy; gianvincenzo.zuccotti@unimi.it; 3 Department of Pediatrics, “V. Buzzi” Children’s Hospital, University of Milan, 20157 Milan, Italy; valeria.calcaterra@unipv.it; 4 Department of Pediatrics, Anesthesiology and Intensive Care Unit, “V. Buzzi” Children’s Hospital, University of Milan, 20157 Milan, Italy; elena.zoia@asst-fbf-sacco.it; 5 “V. Buzzi” Children’s Hospital, ASST Fatebenefratelli Sacco, 20157 Milan, Italy; giuseppe.defilippis@asst-fbf-sacco.it (G.D.F.); alessandro.visconti@asst-fbf-sacco.it (A.V.); 6 Pediatric and Adolescent Unit, Department of Internal Medicine, University of Pavia, 27100 Pavia, Italy

**Keywords:** COVID-19, pediatric surgery, children, emergency

## Abstract

During the coronavirus disease of 2019 (COVID-19) emergency, in the pediatric surgical setting, it has been essential to avoid and contain infections as well as to protect both the patients and the surgical team. During this emergency, procedures and workflow were adapted to provide the safest possible environment for both the surgical team and the patients. Pediatric surgical activities were reorganized during the COVID-19 pandemic at the “Vittore Buzzi” Children’s Hospital, which is a pediatric/maternal hospital located in Milan (Lombardy Region), Italy. Resources were optimized in order to maintain high levels of care and quality of assistance. During the COVID-19 emergency, the pediatric surgical department at the “Vittore Buzzi” Children’s Hospital became an acute care surgical service. For the reorganization of surgical activities, institutional protocols were adapted in order to preserve the pediatric-specific characteristics of our service; five crucial points were specifically addressed. The pediatric surgical procedures carried out during the initial two months of the Italian lockdown are also reported. Continuity of care was maintained for children affected by severe diseases, such as tumors and neurosurgical conditions, whose treatment could not be deferred. Telemedicine and telecommunication were adopted as quick-support modalities for pre- and post-operative care. This reorganization allowed us to preserve the “pediatric specificity” and all care-related procedures offered at this high-quality/high-volume surgical care referral center.

## 1. Introduction

The coronavirus disease of 2019 (COVID-19) outbreak, of which the first case was discovered in late 2019 in Wuhan, China, subsequently spread to other countries and the rest of the world, and was declared a pandemic by the World Health Organization (WHO) on 11 March 2020 [1]. On 20 February 2020, the first case of Severe Acute Respiratory Syndrome Coronavirus 2 (SARS-CoV-2) was confirmed in Italy, in Codogno, northwest Lombardy. The 8 March 2020 is considered the first day of the Italian lockdown, when the Italian government implemented extraordinary measures to limit viral transmission.

The Chinese Centers for Disease Control and Prevention reported that as of 11 February 2020, among the 44,672 confirmed COVID-19 cases, only 416 (0.9%) were people aged 0–10 years and, similarly, a small number (549, 1.2%) were people between 10 and 19 years old [2,3,4]. Likewise, in Italy, as of 18 March 2020, 27 days after the first documented case, only 1.2% of the 22,512 Italian cases with COVID-19 were children, with no reported deaths [5].

A significantly lower proportion of children and adolescents have been affected in comparison with adults, and 90% of these patients were asymptomatic or presented mild or moderate symptoms [5,6,7,8,9,10,11,12,13,14]. The reason for this lower susceptibility is unknown, although several hypotheses have been put forward [2,15,16]: First, children have a more active innate immune response, and this first line of defense against pathogens responds immediately to foreign invaders; the simultaneous presence of other viruses in the mucosa of the lungs and airways, which are common in young children, may also limit the growth of SARS-CoV-2 through direct virus-to-virus interactions and competition; their respiratory tract is healthier because they have not been exposed to as much cigarette smoke and air pollution as adults; and children participate in fewer outdoor activities and undertake less international travel. Moreover, they usually have fewer underlying disorders and stronger self-healing capabilities. Importantly, differences in the distribution, maturation, and functioning of the viral receptor angiotensin-converting enzyme-2 (ACE2) have also been reported.

Here, we share our experience and the pediatric surgical activity reorganization that was implemented during the epidemic at the “Vittore Buzzi” Children’s Hospital, ASST (Azienda Socio-Sanitaria Territoriale, territorial health facility) Fatebenefratelli-Sacco, located in Milan, in the Lombardy Region, the heart of the Italian coronavirus outbreak. Generally speaking, we optimized resources in order to maintain the highest levels of care and quality of assistance while limiting infectious risks for our pediatric patients and their families. However, considering the importance of the association between emergency care and perspective care, we strove not to neglect the primary care of fragile and chronic patients. In the following section, we have detailed the five principal steps that we undertook.

## 2. Five Crucial Reorganization Points

### 2.1. Preservation of Pediatric Specificity during the COVID-19 Pandemic

Anatomical and physiological changes occur during infancy, childhood, and adolescence. Infants and very young children are known to have increased vulnerability to environmental and other injuries because of their size, immature anatomy and physiology, and differing pharmacodynamics. Specific organ development determines the different patterns of disease that occur in childhood and affects drug treatments and responses to them [17,18,19,20]. Therefore, children require age-appropriate pathophysiological and cognitive management, and they should not be considered “little adults” [17]. It is of utmost importance to preserve, even in this period, suitable pediatric treatments for children affected with congenital malformations, tumors, rare diseases, neurological impairment, or acquired surgical pathologies. Professionals with appropriate and dedicated skills are required to guarantee the best quality of care. Starting from the very beginning of the COVID-19 emergency, the pediatric network lost beds, nurses, and doctors at each hospital to support the necessary assistance required to care for the massive number of adult COVID-19 patients. In our pediatric surgical department, dedicated patient management was guaranteed, although all elective surgical interventions, deferrable endoscopic procedures, and outpatient activities were cancelled according to regional and governmental indications and according to the recommendations of the Italian Society of Pediatric Surgery during the COVID-19 Pandemic (disclosed but not published). Patient reception was guaranteed mainly through Emergency Department (ED) access. Although at our hospital, seven pediatric intensive care beds were commandeered to support the regional emergency network for adults, critical pediatric assistance and activities were guaranteed.

### 2.2. Reorganization of Working Spaces and Resources

The reorganization of our hospital building required the union of in-patient wards over three floors instead of the usual five. In this manner, we were able to optimize the distribution of resources and streamline hospital flow. One of the floors was a dedicated maternal ward, one was dedicated to pediatric surgical patients, and one was kept for the neonatal intensive care unit. Pediatric wards were reorganized in another building over two floors; one was entirely dedicated to COVID-19 patients. Intensive care unit (ICU) beds were divided as follows: seven COVID-19 beds in the former ICU ward—six for adults and one pediatric—and four non-COVID-19 beds were created in the surgical unit by converting two of our three operating theaters. Only one operating room was kept for pediatric surgeries.

Our pediatric surgical department was transformed into an acute care department, and triage was based on urgency. The multidisciplinary surgical team included general pediatric surgeons, pediatric orthopedists, a pediatric otolaryngologist, pediatric ophthalmologists, vascular surgeons, neurosurgeons, endoscopists, nursing staff, and caregivers. Patients admitted for surgical procedures had a wide range of pathologies; ages varied from infancy to adolescence, and low and intermediate levels of urgency were included.

Either by slightly exceeding our inpatient reception capacities or by adopting a fast-track system whenever possible, the transfer of children to other general hospitals was avoided. All standards of urgent emergency care were guaranteed, including diagnostic studies and even bedside radiology evaluations, when possible (mainly sonographic studies and abdominal or thoracic X-rays in COVID-19 patients).

A crucial point in the reorganization of our operating theaters (OTs) was the additional amount of time required to disinfect the surgical areas after each procedure. OT sanitation times were estimated to be double those of the pre-pandemic period. Consequently, our OT occupation index increased from 140% to 180% during this period. In addition to this, when the OT space was shared between different surgical specialties, interoperative times also increased due to the labor-intensive movements of instruments and devices specific for each specialty inside and outside the OT.

### 2.3. Definition of Adapted Surgical Protocols

The absence of standardized protocols rendered the initial management of our surgical unit—and, more generally, of the whole hospital—difficult. The Italian Society of Pediatric Surgery proposed recommendations, which were applied in our department. As summarized in Table 1, the recommendations prioritized the preservation and protection of the surgical team and the patients’ health, and provided suggestions on how to streamline the departmental workflow. The list of non-deferrable and deferrable interventions is detailed in Table 1.

### 2.4. Standardization of Phases of Patient Care

To swiftly adapt the department workflow to the numerous changes, we analyzed our behaviors and divided them into “four standardized phases of care”: (1) patient reception; (2) diagnostic workup; (3) therapy; (4) discharge and follow-up. The details of each phase of patient care are described in Table 2.

### 2.5. Additional Supportive Care

Information initiatives included public communications, the creation of a text messaging service, and access to health information at the children’s hospital reception. Informative illustrations regarding the operating unit’s access were planned by our Chief Medical Officer (CMO) in order to reassure families about the risk/benefit ratio of some routine activities that were still offered by the hospital, such as some screening procedures. Families were reassured about the safety protocols that were adopted to avoid the spread of the infection in all hospital areas.

Social distancing requirements were supported by telecommunication with children, families, and pediatricians during the postoperative follow-up for acute diseases. A virtual platform using smartphones and webcam-enabled computers was adopted. This platform was utilized to perform presurgical counseling and to collect anesthesiological and surgical consent, since every patient was accompanied by only one parent/caregiver. Telecommunication was also used during the immediate postoperative period before the child’s discharge to keep the other parent informed and reassured.

Finally, real-time virtual consultation services were created for the continuity of follow-up for chronic and “fragile” patients who, although not always in need of urgent care, require not only permanent monitoring, but also constant dialogue with their hospital interlocutors and frequent reassurance from their healthcare professionals.

## 3. Epidemiological and Surgical Reports

After Italy’s lockdown at the beginning of March 2020, most general hospitals observed an alarming increase in ED admissions [21]. In stark contrast, we detected a significant decrease in ED visits for both medical and surgical issues. As confirmed by recently published national data [22], the decrease was around 80% if we compare the pre-lockdown with the immediate post-lockdown period. This is clearly shown by the differences between a randomly sampled single day (15 February), with a total of 113 visits, and a single day in March (15 March), with only 23 visits over a 24 h period. Moreover, total ED admissions in March 2019 decreased from about 3000 accesses to 600 compared with March 2020.

A proportional increase in ED admissions for domestic accidents (including burns, trauma, and foreign body ingestion) was, however, registered. This phenomenon could be partly attributable to the closure of schools and recreational and sports activities, which, while reducing the rate of acute infections and sport/outdoor traumas, determined an increase in ED admissions for domestic accidents.

We also realized that people were afraid of contracting the infection inside the hospital; thus, a certain number of delayed surgical diagnoses were recorded. Due to this fear by parents and caregivers of being exposed to the contagion by accessing hospitals [22], we registered an increased risk of morbidity even for the most common and recurrent surgical pathologies. Patients presented with more severe clinical manifestations than usually recorded, e.g., severe signs of infections in acute appendicitis or signs of necrosis in most cases of testicular or ovarian torsion. 

During the Italian lockdown from 8 March until 28 April (51 days), in our pediatric surgical unit, we performed 110 procedures on 80 patients, including oncological (*n* = 5), neonatal (*n* = 8), gastrointestinal (*n* = 61), urological (*n* = 10), and other interventions (*n* = 26), as shown in Table 3. The mean age at surgery was 6.6 years (ranging from two days to 15.6 years). All the nasopharyngeal swabs performed on the surgical patients and their caregivers resulted negative. The total number of surgical procedures performed by the different surgical specialties is reported in Table 4. 

Non-elective, non-deferrable surgical activities comprised the management of complex malformations and tumors without sacrificing the quality of surgical care usually guaranteed in the department.

## 4. Conclusions

The reorganization of the pediatric surgical activities during this period at our Children’s Hospital required the adaptation of all surgical protocols and the standardization of the different phases of care. The COVID-19 pandemic compelled the conversion of our pediatric surgical department into an acute care surgical department. These adaptations were feasible thanks to the presence of a multidisciplinary team at different levels of “intensity of care”. The changes necessitated by the COVID-19 emergency most likely modified or worsened the disease course of many patients, with a higher risk of delayed diagnosis and increased morbidity (this should be analyzed in the future). We realize that our efforts to provide a high level of “emergency care” were not adequately accompanied by “perspective care” philosophy, which is a cornerstone of pediatric surgical care. While understanding the extreme gravity of the emergency that has garnered everyone’s attention, we are worried about the loss of “perspective care”, an indispensable component of the management of “fragile” children, e.g., newborns with complex congenital malformations and children/adolescents with chronic pathologies for which the continuity of dedicated care is imperative. For these patients, home isolation and postponed scheduled outpatient appointments could lead to disease aggravation and delays in treatment. Even under emergency conditions, when resources are shifted to critical areas, “pediatric specificity” and all pediatric-care-related particularities should not be overlooked.

## Figures and Tables

**Table 1:** Modified version of the recommendations issued by the Italian Society of Pediatric Surgery during the COVID-19 Pandemic.

**Preservation and Protection of the Team’s Health and Workflow1** Healthcare and all procedures must be carried out with flexibility, through collaboration, and utilizing team management skills in the following ways: alternating surgical roles alternating non-surgical roles staff scheduling that includes “healthy reserves” rest and recovery after all shifts psychological support for the team. It is essential to contain the infection risk of the staff in the following ways: Always apply “non”-surgical solutions whenever possible Use PPE (personal protective equipment) respecting ministerial guidelines and indications All medical and nursing staff engaged in interventions on positive or suspect children must comply with tertiary protection rules, as also indicated by the Ministry of Health and the World Health Organization (WHO).
**Preservation and Protection of Patient Health** The surgical approach must be performed by adhering to the following recommendations: Open surgery: Use of all protective devices required in the event of positive or suspected COVID-19 (visor, protective glasses, mask, FFP2 or N95, High Protection surgical gowns) and suction systems for fume removal are recommended. Minimally invasive surgery (laparoscopy and thoracoscopy): It is absolutely essential to monitor the use of low pressures; the use of filters of the insufflation circuit and the use of closed suction systems are mandatory. Endoscopic procedures: It is recommended to use all protective devices required in the event of positive or suspected COVID-19 (visor, protective glasses, mask, FFP2 or N95, HP gowns), as is the use of appropriate devices for the collection and disposal of fumes.
**Type of patients** (a) Urgent patients: Urgent/non-deferrable patients should be considered COVID-19-positive, unless negative results are available for: rapid reading buffer/antibody dosage and/or lung ultrasound and/or chest Computed Tomography at ultralow doses and with iterative reconstructions. Deferable procedures: Buffer execution for virus research. (b) COVID-19-positive patients: Urgent procedures: The risks/benefits of a minimally invasive approach should be carefully assessed with respect to open surgery, a reason for greater contamination of all staff and the operating room. In the case of intestinal resections and anastomoses with a high risk of dehiscence, perform ostomy. Deferable procedure: Buffer /CT/ intervention with PPE.

**Table 2:** Standardization of the “four phases of care” in a pediatric surgery unit during the COVID-19 health emergency.

**(a) Patient reception phase**
**Reception of the surgical patient through the Emergency Department (ED):** ED tracks were systematically organized by ED pediatricians, starting from the triage phase, into: Blue track or “suspect COVID” (s-COVID) for patients presenting suspicious symptoms (cough, fever, dyspnea, vomiting, diarrhea), with direct access to a protected area, fully furnished with personal protective equipment (PPE) for nurses and doctors, and with the possibility of immediate execution of a nasopharyngeal swab. Red track or “non-suspect-COVID” (ns-COVID) for patients without suspicious symptoms, presenting with other types of pathologies. Each track is managed by dedicated personnel, with appropriate PPE usage regulated by protocols issued by the Health Management Team. Starting from the triage room, each child may be accompanied by a single parent or caregiver with PPE. The patient, either s-COVID or ns-COVID, undergoes a nasopharyngeal swab for COVID; at the same time, a nasopharyngeal swab is also performed on the parent or caregiver who will remain with the patient for the entire hospital stay. Pending the outcome of the swabs, the patient and his/her caregiver are hospitalized in the pediatric surgery ward in single rooms for s-COVID, within a dedicated area identified as the “s-COVID Area”, which is separated from the rest of the ward, which is identified as the “Clean Area”. In the s-COVID area, assistance will be performed using all safety measures defined for SARS-CoV-2-positive (COV+) patients as per institutional, regional, and government recommendations. Upon arrival of the swab reports, the patient and his caregiver will access the definitive accommodation for the remainder of their stay: COV+: Hospitalization will continue in the dedicated inpatient area within the pediatric COV+ wards, which have ad-hoc isolation rooms suitable for the management of infected patients. The patient remains under surgical care or is managed by combined surgical and pediatric care in case of COVID-19 symptoms. COVID-negative (COV–): The patient and his/her caregiver can be transferred to the Clean Area of the pediatric surgical ward.
**Reception of the patient coming from another hospital/other ward:** Each patient without a test for COVID-19 is managed as s-COVID, then subjected to a nasopharyngeal swab together with one parent or caregiver who will remain with him/her for the duration of the hospital stay, and will be temporarily placed in the s-COVID surgical area until the outcome of the swab is available. Access for other family members within the ward is not permitted for s-COVID, COV+, or COV–. The parent who does not have access to the ward can conduct telephone interviews with the doctors.
**(b) Diagnosis phase:** The collection of the patient’s history includes the list of symptoms and epidemiological history (i.e., history of contacts that the patient and his/her parents/siblings have had with positive or suspected individuals) in order to determine a preliminary “risk grading”, which implies different modalities of infection control precautions [9,14,21,22,23]. SARS-CoV-2 swabs are performed as described in the reception phase. Other routine radiological investigations for COVID-19 are not indicated in the absence of symptoms. Within the “s-COVID” or “COV+” areas, radiological investigations are performed at the patient’s bed whenever possible (X-ray, ultrasound), trying to limit the indication to investigations that require moving the patient to the radiology department. In the context of uncertain diagnosis and treatment of patients, frequent multidisciplinary discussion remains the cornerstone of clinical practice, using appropriate modalities, such as online meetings, smaller meetings, and/or telemedicine and teleconsultation.
**(c) Therapy phase** In both the cases of COV+ and s-COVID patients, the operating room must be considered a contaminated environment. Each patient is treated as COV+ unless proven otherwise; all operators (surgeons, anesthesiologists, nurses) must wear appropriate PPE; the technical recommendations already provided by national and international surgical societies and experts should be followed [24,25,26,27].
**(d) Discharge and follow-up phase** The discharge is planned through a combined surgical and pediatric evaluation. In case a patient and/or caregiver tests positive, home quarantine must involve the entire family, considering that the incubation period could have determined the positivity of the other members of the family.Follow-up of the COV+ patient should be postponed to the date of his/her negativization. If this is not possible, the patient must be assessed within a dedicated COV+ area. Follow-up of the COV− patient can be carried out according to standard outpatient procedures, always respecting the protection rules established by the protocols in force.

**Table 3:** Non-elective and non-deferrable pediatric surgical procedures carried out at our pediatric hospital during the Italian lockdown starting from 9 March until 28 April 2020.

	Type of Procedure (Non-Elective, Non-Deferrable)	*n*	Total = 110
Oncologic surgery	Wilms tumor: nephroureterectomy	1	5
	Neuroblastic tumor resection	1	
	Tumor biopsy	1	
	Soft tissue mass excision	1	
	Ovarian mass excision	1	
Neonatal surgery	Laparoscopic pyloromyotomy for pyloric stenosis	2	8
	Bowel resection for meconium peritonitis	2	
	Ladd procedure for neonatal malrotation	1	
	Primary anastomosis for type C esophageal atresia	1	
	Kimura procedure for duodenal stenosis	1	
	Anastomosis for type A esophageal atresia	1	
Gastrointestinal surgery	Endoscopy	24	61
	Laparoscopic appendectomy for non-complicated appendicitis	15	
	Laparoscopic appendectomy for complicated appendicitis	9	
	Bowel obstruction: laparotomy, adhesiolysis, and/or resection	4	
	Replacement of gastrostomy/transgastric jejunostomy	2	
	Decompressive laparotomy in septic shock	2	
	Treatment of hiatal hernia after esophageal atresia correction	1	
	Manual reduction of intussusception	1	
	Resection of cecal duplication	1	
	Total colectomy for toxic megacolon	1	
	Bowel resection for ischemia and perforation after ingestion of magnetic foreign body	1	
Urology	Testicular torsion: derotation and fixation	4	10
	Testicular torsion: orchiectomy	3	
	Circumcision for paraphimosis	1	
	Circumcision for urinary retention	1	
	Cystoscopy for JJ-stent removal (urinary tract infection)	1	
Other procedures	Central venous catheterization	8	26
	Burn dressing	8	
	Treatment of incarcerated inguinal hernia	3	
	Suture of lacerations (one dog bite) *	2	
	Tracheobroncoscopy	1	
	Ovarian torsion and cyst: derotation and cystectomy	1	
	Lymphadenectomy	1	
	Ventriculoperitoneal shunt malfunctioning: revision	1	
	Splenectomy for wandering spleen	1	

* Small non-complicated lacerations were sutured in the Emergency Department.

**Table 4:** Total number of non-elective non-deferrable surgical procedures performed after initiation of the Italian lockdown on March 9 until April 28, 2020 at the ”V. Buzzi” Children’s Hospital, Milan, Italy.

Specialty	N. of Procedures
Pediatric surgery	110
Gynecology	42
Otolaryngology	14
Orthopedics	6
Vascular surgery	1
